# Analyzing the Impact of Different Bonding Protocol Strategies to Improve the Fracture Resistance of Uncomplicated Crown Fractures on Tooth Fracture Reattachment in Permanent Anterior Teeth: An In-vitro Study

**DOI:** 10.7759/cureus.56127

**Published:** 2024-03-13

**Authors:** Vivek D Mahale, Pradeep Solete, Delphine Priscilla Antony, Hima Sandeep Adimulapu, Hema M., Vigneshwar Sambandam

**Affiliations:** 1 Conservative Dentistry and Endodontics, Saveetha Dental College and Hospitals, Saveetha Institute of Medical and Technical Sciences (SIMATS) Saveetha University, Chennai, IND

**Keywords:** total etch, self etch, selective etch, novel, innovative technique, fracture resistance, fractured tooth reattachment, dental

## Abstract

Objective

This study aims to analyze three different bonding protocol strategies in determining the fracture resistance on the reattachment of fragments in permanent anterior teeth.

Methodology

This study evaluated the ability of three bonding methods (Group A, total-etch technique; Group B, selective-etch technique; and Group C, self-etch technique) to enhance the fracture resistance of reattached tooth fragments. Sixty permanent maxillary central incisors were chosen, segmented at 3 mm from the incisal edge, and randomly distributed among the three groups. Tooth fragments were stored for 24 hours in GC Tooth Mousse (GC Corporation, Tokyo, Japan), and then reattachment was done using respective bonding techniques. Fracture resistance was gauged using a universal testing machine.

Results

The mean fracture resistance values were as follows: total-etch (419.5995 N), selective-etch (359.1448 N), and self-etch (192.0887 N). One-way analysis of variance (ANOVA) and post hoc Tukey tests revealed a statistically significant difference between the groups. It was inferred that the total-etch technique exhibited the highest fracture resistance, while the self-etch technique resulted in the lowest fracture resistance (*P* < 0.05).

Conclusions

The choice of bonding technique for reattaching tooth fragments should be made based on clinical context and patient needs. Total-etch provided the highest fracture resistance, but selective etch can be preferred for anterior teeth with lower occlusal loads to prevent sensitivity. The self-etch technique may not provide sufficient strength and should be used cautiously. More clinical studies are required to validate these findings and guide clinical decision-making in traumatic dental injury management.

## Introduction

Dental trauma resulting in an anterior tooth fracture is a common problem that affects children and can have detrimental effects on an individual's psyche and aesthetics [1]. Incisal third fractures of permanent incisors account for 18% to 22% of all injuries to teeth; maxillary incisors account for 96% of these fractures [2]. Dental injuries that occur most frequently are coronal fractures of the anterior teeth [3] and various treatment options have been advocated for the restoration of fractured tooth structures, ranging from conservative approaches such as bonded composite restoration to more extensive options like ceramic crowns [4]. The concept of *fragment reattachment* has emerged in the realm of adhesive dentistry. This method has many benefits, including being extremely conservative and encouraging the preservation of the patient's original tooth structure, as well as being cost-effective and having excellent aesthetics and patient acceptability [5].

The design of the tooth preparation and the degree to which the fragment is firmly bonded to the tooth are important factors that determine the prognosis. For the union of fragmented segments, numerous studies have been conducted employing various storage mediums, restorative materials, and tooth preparation techniques. Out of many storage media used, tooth mousse has shown promising results in enhancing bond strength when stored for 24 hours, since tooth mousse contains a substantial amount of crucial substances like calcium and phosphate [6]. Similarly, in contrast to alternative procedures, the over-contouring technique with the application of nanohybrid resin composites has demonstrated superior outcomes [7]. Nanohybrid composites not only offer aesthetic advantages [8] with a chameleon effect but also contribute to enhanced results [9]. Therefore, to evaluate and compare the effects of various bonding techniques on the fracture resistance of reattached tooth fragments, this study used a combination of over-contoured preparation and nanohybrid composite. The null hypothesis suggests that there is no notable difference in the fracture resistance observed during the reattachment of tooth fragments when assessing different bonding procedures such as total-etch, selective-etch, and self-etch techniques. 

## Materials and methods

Sample size calculation

In a study by Abdulkhayum et al. [10], four different reattachment techniques were evaluated. Based on the mean fracture resistance for the over-contour and external chamfer technique values using G*Power 3.1.9.7, 95% power, a total sample size of 60 participants was determined. The study was approved by the Institutional Ethical Committee (SRB/SDC/ENDO-2203/23/030).

Sample selection

Sixty permanent maxillary central incisors that had to be removed due to periodontal disease were chosen. All samples were examined by a trained operator and were divided into groups using block randomization, with 20 samples per group. Allocation concealment was done in an opaque envelope for all the groups. Ultrasonic scaler tips were used to clean each tooth. Before experimenting, the teeth were treated with a 0.2% thymol solution for disinfection and then kept in distilled water for preservation [11]. To rule out any teeth with abnormalities, they were checked under x21 magnification using a dental operating microscope. Any teeth found with abnormalities were subsequently excluded from the study.

Grouping and Sectioning of Specimens

**Figure 1:**
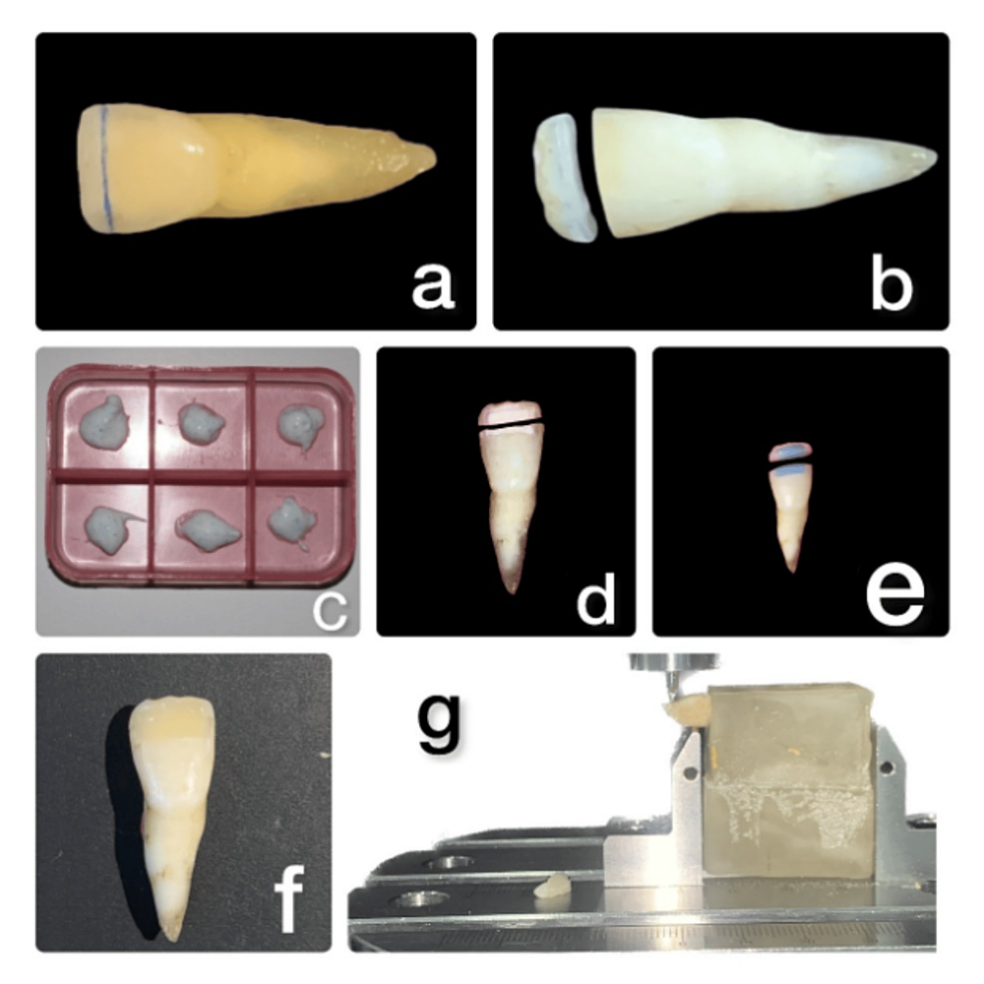
Sample preparation: (a) Marking the specimen for sectioning 3 mm away from the incisal edge; (b) sectioned specimen; (c) sectioned tooth fragments stored in GC Tooth Mousse; (d) over-contoured tooth preparation; (e) etching using 37% phosphoric acid for the total-etch technique; (f) restoration done using a nanohybrid composite; and (g) fracture resistance measured using the universal testing machine.

The following three groups were chosen: Group A, total-etch technique (20 teeth); Group B, selective self-etch technique (20 teeth); and Group C, self-etch technique (20 teeth). The experimental teeth were sectioned 3 mm from the incisal edge using a diamond disc (Figures 1a-1b). Following the sectioning process, the matched fragments were placed in dental mousse (GC Tooth Mousse) for 24 hours at room temperature within pre-labeled trays (Figure 1c). The apical parts of the teeth were kept in distilled water in the meantime. All procedures were performed by a single trained postgraduate student.

Fragment reattachment

Group A

Total-etch adhesives: Over-contoured tooth preparation involved using a straight fissure diamond abrasive, creating an excess of 2.5 mm both coronally and apically from the fracture line at a depth of 0.3 mm on the buccal surface (Figure 1d). Enamel and dentin were etched using 37% phosphoric acid (3M ESPE, St. Paul, MN) followed by gentle drying with cotton pallets (Figure 1e). The adhesive system (3M ESPE, Single Bond Universal, St. Paul) was then applied to both dental pieces using a micro brush. It was then rubbed for 20 seconds after being left undisturbed for 10 to 15 seconds [12]. The adhesive coating underwent air thinning for five seconds. Subsequently, LED light-curing equipment with an intensity of 1,200 mW/cm² was used to light-cure the adhesive layer, following the manufacturer's guidelines. A slightly over-contoured tooth surface was achieved by restoring the buccal surface with multiple increments of nanohybrid composite (Neo Spectra ST, Dentsply Sirona, Shade A2, York, PA) following the application of the adhesive system. 

Group B

Selective-etch technique: Over-contoured tooth preparation was done. Only the enamel was etched with 37% phosphoric acid (3M ESPE) for 15 seconds. After applying the adhesive system (3M ESPE, Single Bond Universal), the nanohybrid composite (Neo Spectra ST, Dentsply Sirona) was added, and the process was completed with light curing for 10 seconds.

Group C

Self-etch technique: Over-contoured tooth preparation was done. Since we are employing a single-step self-etch adhesive, etching was avoided. A bonding agent was applied (3M ESPE, Single Bond Universal) using the same technique, as explained in the previous step, and light cured for 10 seconds, followed by restoring teeth with nanohybrid composite(Neo Spectra ST, Dentsply Sirona) (Figure 1f).

Specimen aging

After reattachment, the teeth were subjected to polishing and finishing using a flexible polishing disc (Shofu Super Snap Mini Kit, Shofu Dental, Ratingen, Germany). Subsequently, they were stored in artificial saliva for 48 hours within a temperature-controlled incubator set at 37 °C. Following this initial phase, the specimens underwent 500 cycles of thermocycling between 5 and 55 °C [5] at the white lab facility of Saveetha Dental College and Hospitals in Chennai.

Measuring fracture resistance

The evaluation of specimen fracture strength was conducted utilizing a universal testing machine (UTM). A chisel is characterized by a 0.5 mm cross-section and a crosshead speed of 1 mm/minute. Each specimen's fragmentation force was measured in Newton (Figure 1g).

Statistical analysis 

Mean and standard deviation values were calculated. Shapiro-Wilk and Kolmogorov-Smirnov tests were conducted to confirm parametric (normal) distribution. A one-way ANOVA was executed to compare multiple groups from independent populations, followed by the Tukey post hoc test. A significance level of *P* < 0.05 was utilized. The statistical analysis was carried out using IBM SPSS Statistics for Windows, Version 22.0 (IBM Corp., Armonk, NY).

## Results

**Figure 2:**
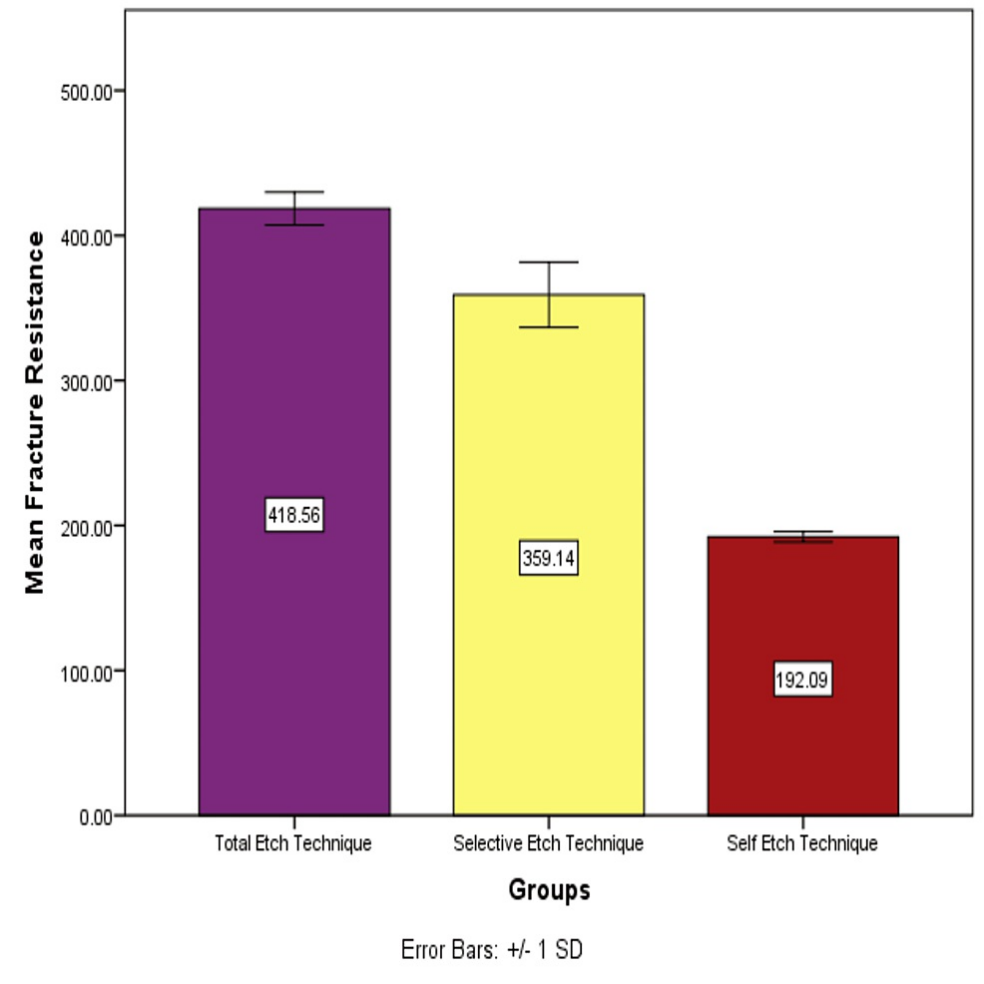
Mean and standard deviation (SD) values for the fracture resistance (N) of three groups. Group A (total-etch technique) showed a statistically significant difference compared to Group B (selective-etch) (*P *< 0.001) and Group C (self-etch technique) (*P *< 0.001). Group B showed a significant difference compared to Group C (*P *< 0.001).

The findings demonstrated significant differences in mean fracture resistance among the three techniques. Hence, the null hypothesis was rejected, stating that a significant difference was observed when different bonding protocol strategies were used. The total-etch technique showed the highest mean fracture resistance of 419.5995 N (Table 1 and Figure 2), whereas the selective-etch technique showed a slightly lower mean fracture resistance of 359.1448 N, and the self-etch technique showed the least mean fracture resistance. The self-etch technique exhibited the lowest mean fracture resistance of 192.0887 N. One-way ANOVA and post hoc tests were carried out. Group A showed a statistically significant difference compared to Group B (*P *< 0.001) and Group C (*P *< 0.001). Group B showed a significant difference compared to Group C (*P *< 0.001).

**Table 1:** Mean and standard deviation (SD) values for the fracture resistance (N) of three groups. Group A (total-etch technique) had the highest mean value, while Group C (self-etch technique) had the lowest mean value.

Groups	N	Mean	Standard deviation
Group A, total-etch technique	20	418.5595	11.29902
Group B, selective-etch technique	20	359.1448	22.54602
Group C, self-etch technique	20	192.0867	3.57182

## Discussion

Dental trauma often leads to the unfortunate fracture of anterior teeth, bringing about detrimental consequences that extend beyond mere dentition damage, encompassing psychological repercussions for the affected patients [13]. The study systematically compared three tooth reattachment techniques such as total-etch, selective-etch, and self-etch in terms of their fracture resistance for anterior teeth.

Several methods are used for fracture reattachment:

1) Simple reattachment: No supplementary preparations are done before bonding fractured fragments [14].

2) External chamfer: Following the reattachment of fractured tooth fragments, a chamfer with a depth of 1.0 mm should be introduced along the fracture line, employing a round bur [15].

3) Over contour: Before reattaching the broken tooth fragments, preparation of the buccal surface is performed using a cylindrical diamond finishing bur. The preparation extends 2.5 mm both above and below the fracture line, with a depth of 0.3 mm. After applying the adhesive system, a resin composite increment is used to reconstruct the buccal surface, creating a tooth surface that is slightly over-contoured. This approach is used in our investigation [10].

4) Internal dentinal groove: Before reattaching the broken tooth fragments, an internal groove with dimensions of 1 mm in depth and 1 mm in width is generated in both the fragment and the remaining tooth. Application of the adhesive system is carried out on each surface. Before commencing light curing, resin composite is inserted into the groove. Following this, the fragment is reattached, and any surplus composite is eliminated. Each surface is subsequently subjected to light curing for 40 seconds [16].

However, the over-contoured preparation approach stands out among these with numerous advantages. It proves to be the most conservative technique, offering optimal potential for cosmetic recovery by replicating the natural tooth's color, contour, surface texture, and translucency. Moreover, the color stability and wear characteristics align closely with those of the surrounding natural teeth [17]. According to a study by Abdulkhayum et al., it was concluded that over-contour showed better performance than other techniques [10].

The total-etch technique emerged as the standout performer in terms of fracture resistance, boasting a mean value of 419.5995 N (Table 1). This method involves the meticulous process of etching both enamel and dentin [18], facilitating the creation of a robust bond between the reattached tooth fragment and the existing tooth structure. This finding aligns with the expectation that total etching can create a more robust bond between the tooth fragment and the tooth structure. The etching process allows for better surface adhesion and potentially greater interlocking of the composite material, contributing to enhanced fracture resistance. The predominantly mixed failure mode in this group, involving both the bonded interface and apical tooth structure, further supports the idea of strong bonding. This technique's effectiveness in providing superior fracture resistance makes it a viable option, especially when maximum strength is required [19]. This technique, with its superior fracture resistance, establishes itself as a viable option, particularly when maximum strength is a critical requirement.

In the middle ground, the selective-etch technique yielded an intermediate mean fracture resistance value of 359.1448 N. While it fell short of the fracture resistance demonstrated by the total-etch technique, it notably outperformed the self-etch technique. The slightly lower fracture resistance, when compared to total etching, may be deemed acceptable, especially for anterior teeth characterized by lower occlusal loads. The selective-etch technique emerges as a practical choice, navigating the delicate balance between strength and application convenience. On the lower end of the spectrum, the self-etch technique exhibited the lowest mean fracture resistance at 192.0887 N. This technique, relying on a self-etch adhesive system without the traditional etching process, displayed a noticeable compromise in bonding strength for reattached tooth fragments. While the self-etch technique offers simplicity in its application, this study suggests that its compromise on structural integrity may limit its suitability, especially when compared to the other two techniques. The selective-etch technique, involving the etching of only enamel [20], yielded intermediate fracture resistance results. Although the mean fracture resistance was lower than that observed with the total-etch technique, it proved significantly higher than the fracture resistance associated with the self-etch technique. This technique may offer a compromise between bonding strength and ease of application. Selective etching retains some of the benefits of total etching while simplifying the procedure. Importantly, the difference in fracture resistance between the total-etch and selective-etch techniques was relatively small. For anterior teeth, where occlusal loads are generally lower than posterior teeth and to address the concern of postoperative sensitivity [20], the selective-etch technique may be a favorable choice. The slightly lower fracture resistance compared to the total-etch technique may not be a significant clinical drawback in these cases. Moreover, the predominant adhesive failure mode suggests that the bond at the interface is less likely to result in catastrophic failures, which can be important in anterior teeth. The self-etch technique, which avoids etching and relies on a self-etch adhesive system, resulted in the lowest mean fracture resistance. This technique's significantly lower fracture resistance values indicate that it may not provide sufficient bonding strength for reattached tooth fragments, particularly when compared to the other two techniques. The prevalence of adhesive failure at the bonded interface further emphasizes the potential vulnerability of the bond in this group. While the self-etch technique offers simplicity and convenience, it may compromise the structural integrity of the reattached fragment. Clinicians should carefully consider the selection of the bonding technique based on the clinical scenario and patient preferences when performing tooth fragment reattachment procedures. The findings of this study emphasize the importance of achieving a strong and durable bond between the tooth fragment and the remaining tooth structure. Although the total-etch technique demonstrated superior resistance to fracture, it may not always be the most suitable choice, particularly when prioritizing simplicity and efficiency. It is advisable to store the fragment in a medium rich in calcium before reattachment, as it can further enhance the bond strength of the reattached fragment. In this study, GC Tooth Mousse was used due to its high concentration of essential elements like calcium and phosphate [21]. Moreover, in a study by Jalannavar and Tavargeri, Tooth Mousse was identified as an effective storage medium for fragment reattachment, leading to increased bond strength [6].

However, it is important to recognize a limitation in this study. Sectioning of the specimen was done using a diamond disc. As a result, the surface of these specimens will be different from a natural fracture because of the presence of a smear layer, which is absent in normal fractures. The inherent fit of naturally occurring fracture pieces is also compromised in disc-cut specimens, making the precise alignment of the tooth and fragment challenging [22]. The Instron Universal Testing Machine E-3000 is employed for assessing the fracture resistance of a designated test specimen. This versatile machine is named for its capacity to conduct a diverse array of tests across various materials by applying tensile, compressive, or transverse stresses [22]. Scanning electron microscopy (SEM) analysis for the fractured segment was not carried out.

Despite these limitations, the present investigation establishes a constant, standardized, and reproducible scenario crucial for in vitro research, providing valuable insights into the evaluated bonding techniques. The fracture resistance results distinctly highlight significant differences among the three techniques. The mean fracture resistance values rank as follows: 419.5995 N for the total-etch technique, 359.1448 N for the selective-etch technique, and 192.0887 N for the self-etch technique. Notably, the total-etch technique exhibits the highest fracture resistance, followed by the selective-etch technique, while the self-etch technique demonstrates the lowest fracture resistance.

Additional research and clinical studies are essential to validate these findings and evaluate the long-term success and clinical applicability of reattaching tooth fragments using different bonding techniques [23]. Understanding the strengths and limitations of each approach will aid dental professionals in providing effective and aesthetically pleasing treatments for traumatic dental injuries, ultimately benefiting patients' oral health and well-being.

## Conclusions

In conclusion, this in vitro study systematically evaluated the fracture resistance of reattached tooth fragments using three different bonding techniques: total-etch, selective-etch, and self-etch. The findings highlighted notable distinctions among these techniques, with the total-etch technique demonstrating the highest fracture resistance, followed by the selective-etch technique, and the self-etch technique showing the lowest fracture resistance. While the total-etch technique offers superior bonding strength, the selective-etch technique may provide a reasonable compromise between strength and simplicity, particularly suitable for anterior teeth with lower occlusal loads. Conversely, the self-etch technique exhibited the lowest fracture resistance and may not be recommended when maximum strength is required. 
